# Supplementary material for: Genotyping of Circulating Free DNA Enables Monitoring of Tumor Dynamics in Synovial Sarcomas
Source: Cancers (Basel). 2022 Apr 21;14(9):2078. doi: 10.3390/cancers14092078 (PMC9105697; doi:10.3390/cancers14092078)
Supplement: Supplementary file 1 [file cancers-14-02078-s001.zip › cancers-1681946-supplementary.pdf]

Supplementary Materials: Genotyping of Circulating Free DNA Enables Monitoring of Tumor Dynamics in Synovial Sarcomas

Anja Eisenhardt, Zacharias Brugger, Ute Lausch, Jurij Kiefer, Johannes Zeller, Alexander Runkel, Adrian Schmid, Peter Bronsert, Julius Wehrle, Andreas Leithner, Bernadette Liegl-Atzwanger, Riccardo E. Giunta, Steffen U. Eisenhardt and David Braig

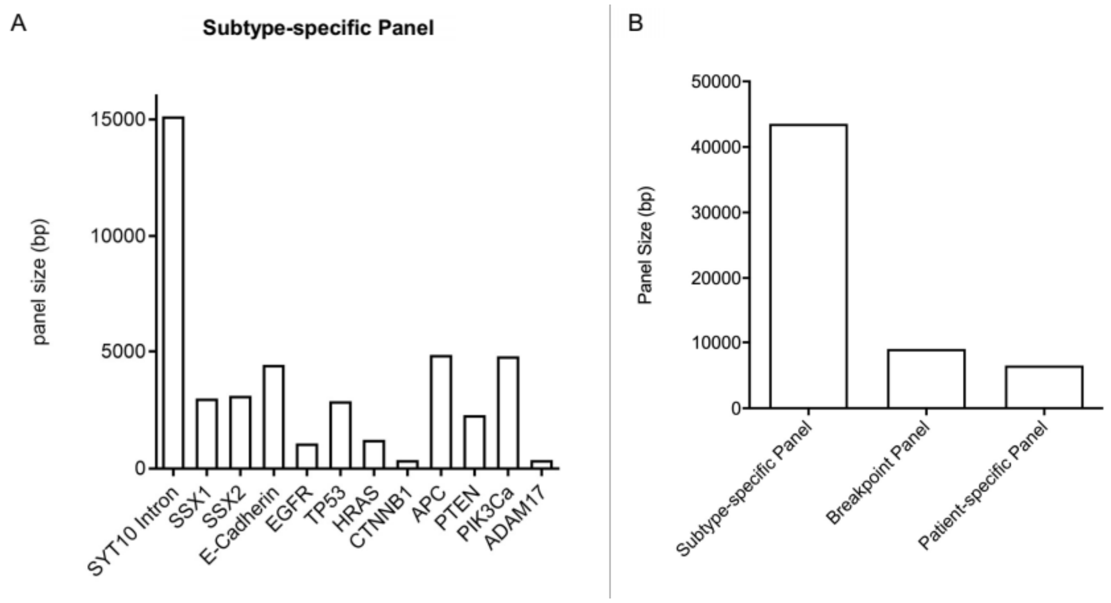

Figure S1. Specifications of enrichment panels. **A**. Composition of the subtype-specific panel. The panel is composed of the regions where the SYT and SSX1/SSX2 breakpoints occur and exonic regions from nine genes, which are reported to harbor mutations in at least 5% of SS tumors**. B.** Panel sizes of the subtype-specific panel, breakpoint panel and patient-specific panel.

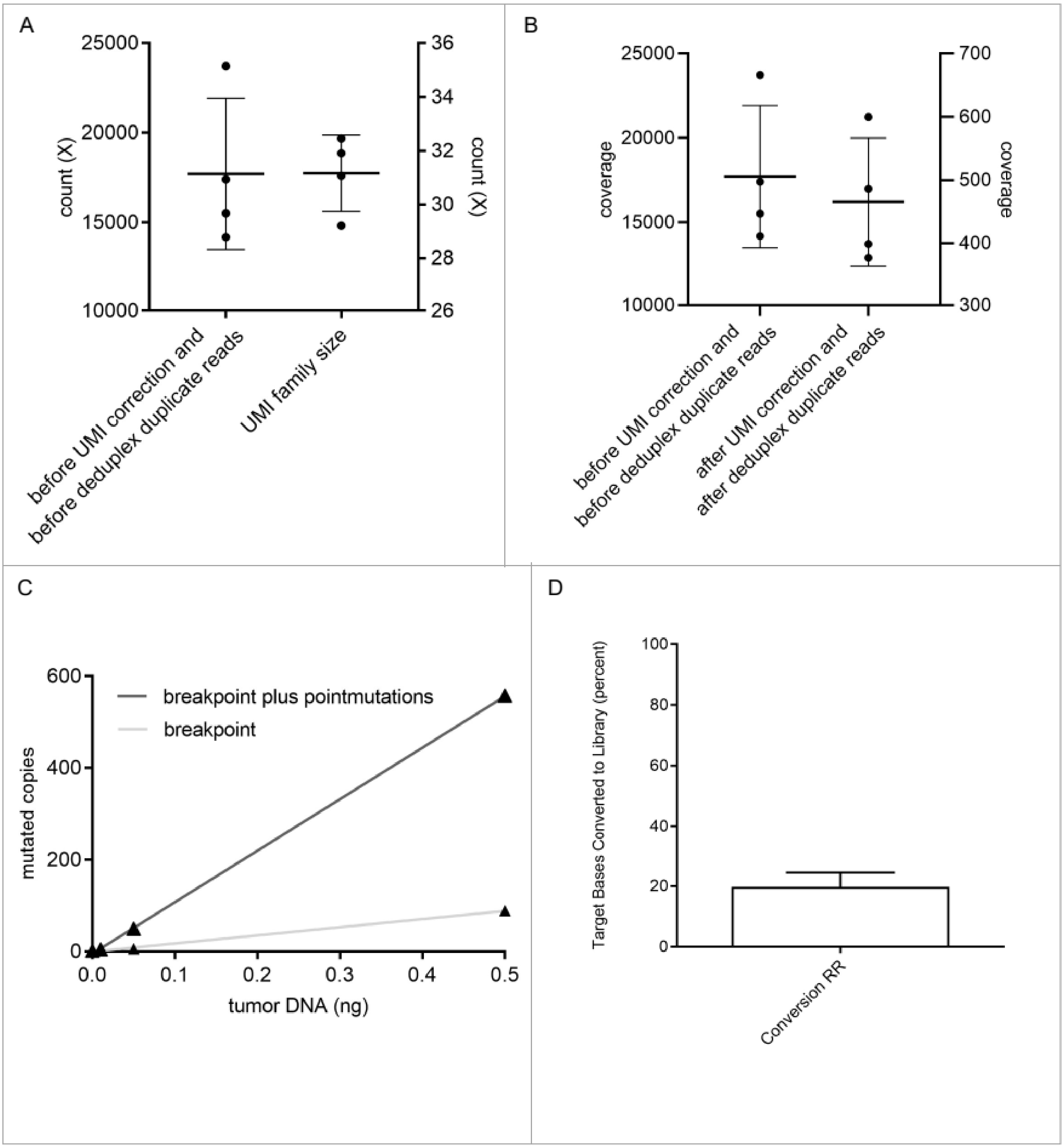

Figure S2. Specifications of the Fuji cell line dilution series. **A**. The left y-axis depicts the total mean coverage before de-duplication or UMI family consensus calling for each sample. On the right y-axis the mean number of reads for each UMI family are denoted (n = 4). **B**. Left Y axis is identical to A. and right y-axis depicts the coverage after de-duplication (n = 4). **C**. Depicts the detected reads for each dilution sample depending if only breakpoint reads or breakpoint and point mutation reads are called. Sensitivity was superior for combined calling of breakpoint and point mutations (n = 4). **D.** Conversion rate of template cfDNA to sequenced library (n = 4). Bars indicate the mean and SD in all subfigures.
